# STAT3 is required but not sufficient for EGF receptor-mediated migration and invasion of human prostate carcinoma cell lines

**DOI:** 10.1038/sj.bjc.6603234

**Published:** 2006-06-27

**Authors:** W Zhou, J R Grandis, A Wells

**Affiliations:** 1 Laboratory and Pathology Service, Pittsburgh VAMC, PA, USA; 2 Department of Pathology, University of Pittsburgh, Scaife Hall, S-713, 3550 Terrace St, Pittsburgh, PA 15261, USA; 3 Department of Otolaryngology, University of Pittsburgh, Pittsburgh, PA 15261 USA

**Keywords:** STAT3, EGF, motility, prostate cancer

## Abstract

Growth factor-induced migration is a rate-limiting step in tumour invasiveness. The molecules that regulate this cellular behaviour would represent novel targets for limiting tumour cell progression. Epidermal growth factor (EGF) receptor (EGFR)-mediated motility, present in both autocrine and paracrine modes in prostate carcinomas, requires *de novo* transcription to persist over times greater than a few hours. Therefore, we sought to define specific signalling pathways that directly alter cellular transcription. Signal transducer and activator of transcription 3 (STAT3) is activated, as determined by electrophoretic motility shift assays, by EGFR in DU145 and PC3 human prostate carcinoma cells in addition to the motility model NR6 fibroblast cell line. Inhibition of STAT3 activity by antisense or siRNA downregulation or expression of a dominant-negative construct limited cell motility as determined by an *in vitro* wound healing assay and invasiveness through a extracellular matrix barrier. The expression of constitutively activated STAT3 did not increase the migration, which indicates that STAT3 is necessary but not sufficient for EGFR-mediated migration. These findings suggest that STAT3 signalling may be a new target for limiting prostate tumour cell invasion. In a microarray gene analysis of what transcription units are altered by EGF in a STAT3-dependent manner we found that the expression of motility-limiting VASP protein and the apoptosis nexus caspase 3 were both downregulated upon EGF exposure. These findings suggest a molecular basis for the STAT3 dependence of EGFR-mediated prostate tumour progression.

Prostate cancer is the single most common form of solid tumour in humans, present in more than 9 million men and is second only to lung cancer in annual cancer deaths of US men (Landis *et al*, 1999). Invasiveness into the adnexa by extension of the primary tumour through the encapsulating matrix and musculature is a major cause of morbidity and mortality or prostate cancer. This invasiveness appears to be due to dysregulated motility (Wells *et al*, 2002; Nguyen and Schrump, 2004). A subset of invasive cells in the primary tumour must recognise and interact with the surrounding extracellular matrix first, and then degrade or remodel the ECM, and finally to migrate through it to reach adjacent tissue. It was demonstrated that integrin-supported motilities enables tumour invasion but growth factor-induced motility promotes it (Rabinovitz and Mercurio, 1996; Gladson, 1999; Kassis *et al*, 2001). Thus, factors that enable this motility would be points of intervention.

The epidermal growth factor receptor (EGFR) lies at the head of a complex signal transduction cascade that contributes to a number of processes important to cancer development and progression. EGF-induced cell motility is important for tumour invasion. In addition to a number of epigenetic signalling events, it was found in fibroblasts that *de novo* transcription is required for motility (Chen *et al*, 1994). One candidate pathway is that of STAT (signal transducer and activator of transcription) proteins, a subset of which are directly activated by EGFR independent of JAK signalling or bridging (David *et al*, 1996). Seven mammalian STAT genes have been found (STAT1, 2, 3, 4, 5a, 5b and 6) that are structurally conserved. Full-length EGFR binds and directly activates signal transducer and activator of transcription 1 (STAT1) and STAT3 (David *et al*, 1996); these function as homo- and heterodimers (Stancito *et al*, 1996; Sato *et al*, 1997). These two STATs have been considered repressors and promoters of carcinogenic growth, respectively.

STAT3 activation is implicated in tumour invasion in head and neck squamous cell and other carcinomas (Grandis *et al*, 1998; Song and Grandis, 2000). A significant correlation has been reported between the expression of nuclear STAT3 and breast cancer as compared to normal mammary tissues (Berclaz *et al*, 2001). Interestingly, prostate tumour cells have been found to contain constitutively activated STAT3, and blockade of this activated STAT3 significantly suppressing the tumour cell growth (Ni *et al*, 2000; Lee *et al*, 2004; Gao *et al*, 2005). This is of key interest as a previous study already had demonstrated that constitutively activated STAT3 can mediate cellular transformation (Bromberg *et al*, 1999). A survey of organ-confined prostate biopsies demonstrated a correlation between local aggressiveness and phospho-STAT3 staining (Horinaga *et al*, 2005). These reports support hypothesizing that STAT3 signalling contributes to carcinogenic progression, in addition to merely increasing the cell number.

Herein, we report that STAT3 signalling downstream from EGFR autocrine activation leads to increase tumour cell motility and invasiveness. Antisense and siRNA downregulation of STAT3 abrogated EGFR-mediated transcription signalling as evinced by inhibition of electrophoretic mobility shift assay (EMSA) shift detection. These interventions also blocked EGFR-mediated fibroblast and DU145 and PC3 human prostate tumour cell migration and transmigration of an ECM barrier, decreasing it to a background level. Use of STAT3 dominant-negative (DN) and constitutive active mutants in fibroblast and human prostate tumour cells provided results consistent with the antisense approach and demonstrated that while STAT3 signalling is required it is not sufficient for the migratory and invasiveness phenotype. Preliminary data on microarray and immunoblot demonstrate a potential relationship between EGFR/STAT3 signalling pathway and the expression of ena/VASP and caspase 3, which related to cell motility and apoptosis, both key processes in tumour progression.

## MATERIALS AND METHODS

### Cell lines and reagents

Human DU145 prostate carcinoma cells (Stone *et al*, 1978) overexpressing EGFR, DU145WT (Xie *et al*, 1995), were maintained in DMEM supplemented with 10% FBS, L-glutamine (2 mM), nonessential amino acids (0.1 mM), sodium pyruvate (1 mM) and 100 U ml^−1^ of penicillin; 350 mg ml^−1^ of G418 was added to the medium for DU145WT cells. Human PC3 prostate cancer cells (Kaighn *et al*, 1979) were maintained in F-12 medium supplemented with 10% FBS, L-glutamine (2 mM), nonessential amino acids (0.1 mM), and sodium pyruvate (1 mM). Both prostate carcinoma cell lines present active autocrine stimulatory loops involving EGFR signalling, as is typical for *de novo* human prostate carcinomas (Wells, 2000); we minimise this effect *in vitro* by altering culture conditions, although this cannot be negated during the invasion studies (Xie *et al*, 1995). For the biochemical studies, NR6WT mouse fibroblasts expressing human EGFR (Wells *et al*, 1990) were maintained in Eagle's medium supplemented with 10% FBS, L-glutamine (2 mM), nonessential amino acids (0.1 mM), sodium pyruvate (1 mM), 100 U ml^−1^ of penicillin and 350 mg ml^−1^ of G418.

### Oligonucleotides and transfections

STAT3 antisense oligonucleotides (Human: 5′-CCA TTG GGC CAT CCT GTT TCT-3′, mouse: 5′-GTT CCA CTG AGC CAT CCT GC-3′) were synthesised with phosphorothieate modification by DNA synthesis facility of the University of Pittsburgh. SiRNA against human STAT3 (5′-GGAGCAGCACCUUCAGGAUTT-3′), mouse STAT3 (5′-UGCAUGUCUCCUUGGCUCUUGAGGG-3′) and eGFP (5′-ACCCGCGCCGAGGUGAAGTT-3′) were from IDT (Skokie, IL, USA). Transfections used the Lipofectamine 2000 method (Life Technologies Inc). Cells were grown in antibiotic-free medium till 50–60% confluence and transfected with siRNA for 24 h. The final concentration of siRNA in the experiment is 100 nM. After transfection, the cells were allowed to recover in antibiotic free medium for 24 h before further experimentation. For these experiments, the ‘no treatment’ control underwent mock transfection (no DNA added), the ‘control’ DNA was that from the other species (e.g. the mouse sequence in human cells). From earlier studies using eGFP, we found that this procedure resulted in >85% of the prostate caricnoma cells expressing the eGFP plasmid (Mamoune *et al*, 2004).

### Nuclear extract preparation and EMSA

For STAT3 analysis by EMSA (Sen and Baltimore, 1986), confluent cells were harvested and washed with ice cold 1 × PBS, with the pellets resuspended in five-fold buffer A (10 mM HEPES, pH 7.9; 1.5 mM MgCl_2_; 10 mM KCl; 1 mM NaF; 0.5 mM DTT; 0.2 mM PMSF; 1 *μ*g *μ*l^−1^ popftain; 5 *μ*g *μ*l^−1^ aprotinin; 2 *μ*g *μ*l^−1^ leupeptin). After incubating on ice for 15 min, the nuclei were centrifuged at 4°C for 10 s. Pellets were resuspended in five-fold volumes (20 *μ*l–100 *μ*l) cold buffer C (20 mM HEPES, pH 7.9; 1.5 mM MgCl_2_; 420 mM NaCl; 10 mM NaF; 1 mM Na_3_VO_4_; 25% glycerol; 0.2 mM EDTA; 0.5 mM DTT; 0.2 mM PMSF; 1 *μ*g *μ*l^−1^ popftain; 5 *μ*g *μ*l^−1^ aprotinin; 2 *μ*g *μ*l^−1^ leupeptin), incubated on ice for 30 min and microcentrifuged for 2 min at the maximum speed. Aliquots were stored at −80°C. For EMSA, 10 *μ*g of total extractions were used for each experimental point. EMSA was performed using a *γ*-^32^P-labelled double-strand oligonucleotide probe m67 (sense: 5′-GAT TTC CCG TAA ATC AT-3′) that binds STAT3 and STAT1 proteins. Protein–DNA complexes were resolved by nondenaturing PAGE gel and detected by autoradiography. Anti-STAT3 antibodies (Upstate Biotechnology, Waltham, MA, USA) were used for super shift assay and a 50-fold excess of unlabelled m67 probe was used in cold competition assay.

### Migration assay

*In vitro* wound healing assay was used to assess cell motility in two dimensions. Cells were plated on a 12-well plate and grown to confluence in their regular medium. Confluent cells were quiesced in 1% dialysed FBS for 24 h before each experiment. A rubber policeman was used to create a denuded area. Cells were washed twice with PBS and treated with or without specific effectors for 24 h. Photographs were taken at hours 0 and 24, and the distance traveled was determined by subtracting the values obtained at hour 0 from 24. Mitomycin C (0.5 *μ*M) was added to the medium to prevent the confounding issue of cell proliferation. The ‘no treatment’ challenge was used to normalize each experiment and is notated as a fractional level of 1.0.

### Invasion assays

Invasive potential was determined *in vitro* by transmigration of an ECM (Niedbala *et al*, 1985). Matrigel invasion chamber plates were purchased from Becton Dickinson/Biocoat (Bedford, MA, USA). The upper surface of the matrix was challenged with 1.5 × 10^4^ cells, a number derived from empirical experimentation. Cells were kept in serum-free medium containing 1% BSA for the first 24 h and then replaced with only serum-free medium for the remaining 24 h; the lower chamber contained medium containing 10% serum for the entire assay. Enumeration of the cells that invaded through the matrix over a 48 h period was accomplished by visually counting cells on the bottom of the filter, as per routine procedures, after any uninvaded cells were removed from the top of the filter with a cotton swab. In all of the cases, individual experiments were performed in duplicate chambers. The ‘no treatment’ challenge was used to normalize each experiment and is notated as a fractional level of 1.0.

### Immunoblotting

Protein expression was determined by immunoblotting using standard means. Confluent cells were washed with ice cold 1 × PBS twice followed by treated with lysis buffer (100 mM Tris-HCl, pH 6.8; 4% SDS; 20% glycerol and 5% B-mercapto-ethanol). Sample proteins were denatured at 100°C for 5 min before loading to the gel. After electrophoresis, the proteins were transferred to a Immobilon-P membrane. Membranes were blocked by 1% BSA. After blocking, membranes were incubated with a primary antibody: mouse anti-STAT3 (Zymed, San Francisco, CA, USA); mouse anti-GAPDH (abCam, Cambridge, MA, USA); mouse anti-Flag (Stratagene); rabbit anti-phosphor-STAT3 (Cell Signaling, Danvers, MA, USA) and rabbit anti-HA-tag (Cell Signaling) at 4°C for overnight or at room temperature for 1 h. The membranes were washed twice with 0.5% Tween-20 (TBST) before incubated with secondary antibodies (Goat anti-mouse Ig or Goat anti-rabbit Ig, Biosource, Carlsbad, CA, USA) and detection as per standard procedures.

### Cell counting

The cells proliferation and toxicity assay were preformed by direct cell enumeration. A Z-series Coulter Counter (Coulter Corp., Miami, FL, USA) was used for the counting. Cells were digested by Tripsin-EDTA (GiBco, Carlsbad, CA, USA) and the resuspended in the original medium followed by a 1 : 100 dilution in Isoton II solution (Beckman, Fullerton, CA, USA) for counting.

## RESULTS

### EGF activates STAT3 in fibroblasts and prostate carcinoma cells

EGFR signalling activates STAT proteins in fibroblasts (David *et al*, 1996) and a variety of carcinoma cells (Grandis *et al*, 1998; Garcia and Jove, 1998; Bowman *et al*, 2000). As we have previously shown that prostate carcinoma cells present autocrine EGFR signalling, we determined whether this also invoked STAT3 activation. To study this issue, we used the motility model system, murine NR6WT fibroblasts, to examine EGF-triggered events as autocrine signalling is absent in these cells in contrast to prostate carcinoma cells which present autocrine EGFR-activating signalling loops, and thus the role of EGFR signalling can be cleanly parsed. The two human prostate cancer cell lines, DU145WT and PC3, present autocrine activation of endogenous and exogenous EGFR, as is the norm for prostate carcinoma cells. However, to parse the function of EGFR signalling, we tested our prostate cells under conditions that minimise autocrine activation (Xie *et al*, 1995).

**Figure 1:**
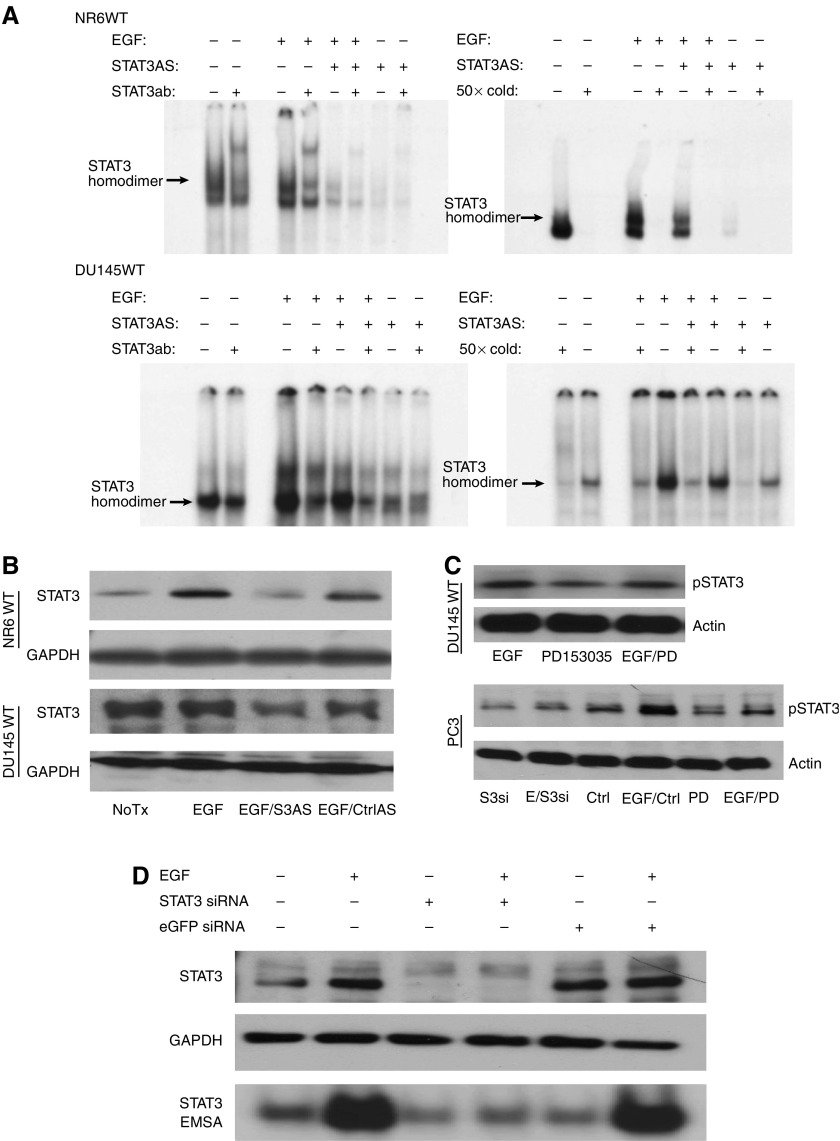
EGF signalling induces STAT3 activity. (**A**) EMSA detects EGF-dependent STAT3 activity in NR6WT fibroblasts and DU145WT prostate tumour cells. EMSA analysis of STAT3 DNA-binding activity in nuclear extracts prepared from mouse fibroblast cells and human prostate tumour cells using the *γ*-P^32^-labelled m67 probe. Supershift with the STAT3 antibody shows the specificity of the STAT3 protein and cold competition demonstrates the specificity of the m67 probe. Cells were treated with 10 nM EGF, 10 nM EGF plus 10 *μ*M STAT3 antisense or 10 *μ*M STAT3 antisense alone (cells were treated 14–18 h). Lower concentrations of cold competition assays (10 × and 5 ×) were also preformed and showed the similar trending results (data not shown). (**B**) Immunoblotting shows that EGF-mediated increase of STAT3 expression is suppressed by STAT3 antisense oligonucleotides, but not altered by nonspecific oligonucleotides. (**C**) Immunoblotting shows that PD153035, an selective inhibitor of EGFR kinase, blocks the activation of STAT3 induced by EGF in both DU145WT and PC3 cells. Also shown for in PC3 cells is that STAT3 siRNA suppresses the activity of STAT3. (**D**) EGF-mediated increase of STAT3 expression and activity is suppressed by STAT3 siRNA in DU145WT cells, but not by random siRNA (against eGFP). The STAT3 siRNA also negated EGF-induced STAT3 activity in DU145WT prostate tumour cells. Similar results were found with PC3 cells. Shown are representative blots of experiments each performed at least three times.

In murine fibroblasts, NR6WT cells, EGF exposure increased EMSA detection of the STAT3 band, which was upshifted upon addition of an antibody to murine STAT3 (Figure 1A). In DU145WT human prostate carcinoma cell lines, EGF increased the STAT3-targeted band; herein the antibody to human STAT3 eliminated DNA binding (Figure 1A). We downregulated the protein using antisense oligonucleotides or siRNA directed against STAT3; these interventions reduced whole cell levels of STAT3 significantly in both NR6WT fibroblasts and DU145WT cells (Figure 1B and D). Antisense and siRNA downregulation reduced the EMSA detection in these cells. Similar findings were noted with PC3 cells (data not shown). Further demonstrating the role of EGFR signalling, the EGFR selective inhibitor PD153035 decreases the effect of exogenous EGF on phosphorylation of STAT3 (Figure 1C); that this was not totally abrogated is due to the limitations of using the inhibitor at concentrations that are nontoxic. These findings demonstrate that STAT3 signalling is present in prostate carcinoma cell lines in response to EGFR signalling and that this signalling pathway increases STAT3 binding to target.

### STAT3 is required for EGFR-mediated fibroblast and human prostate tumour cell motility and invasiveness

**Figure 2:**
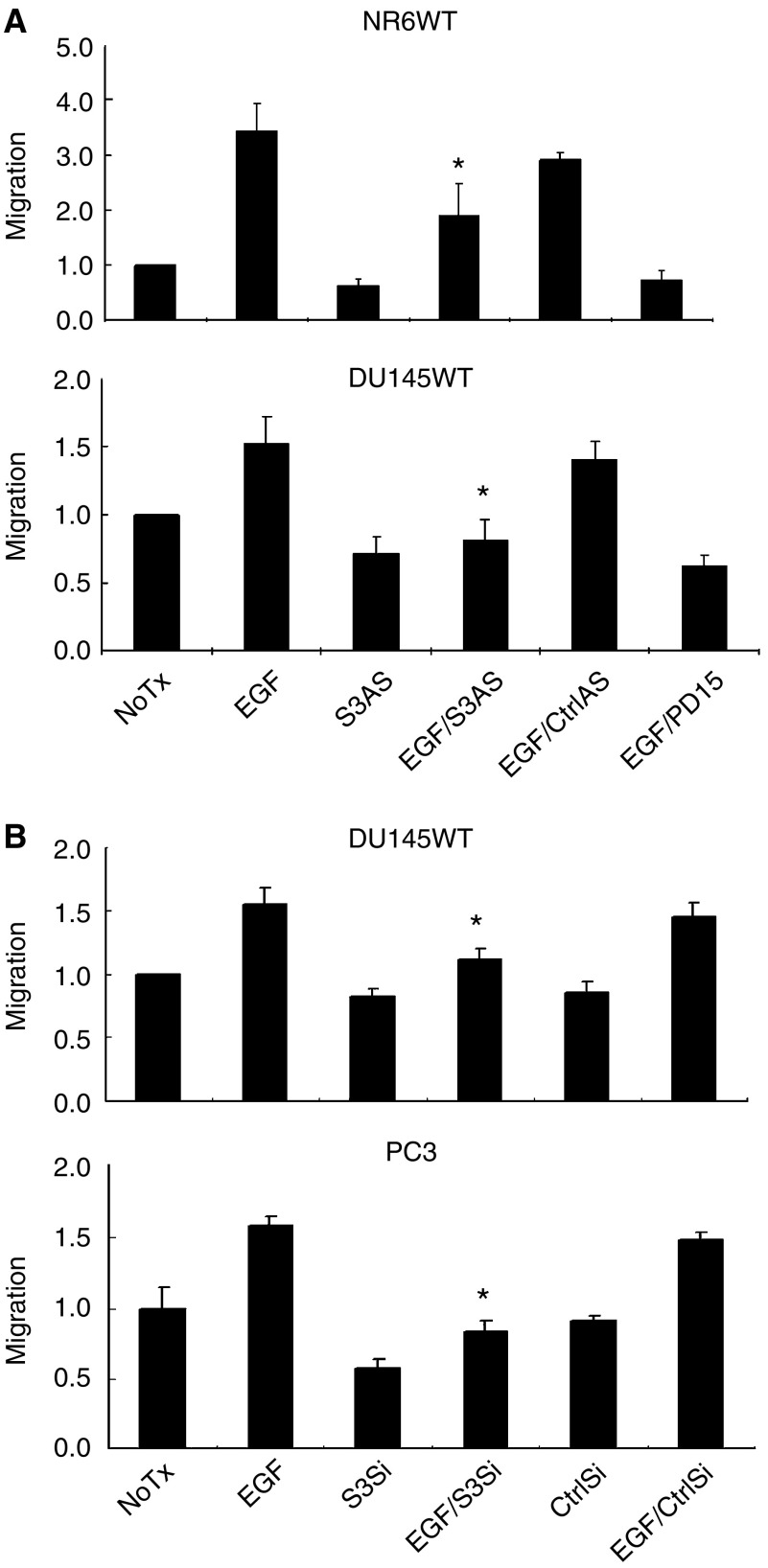
STAT3 is required for EGF-receptor-mediated cell migration in mouse fibroblast cells and human prostate tumour cells. (**A**) The migration distance of NR6WT fibroblast and DU145WT prostate tumour cells after treated with 1 nM EGF for 24 h was significantly greater and this increase was suppressed by 10 *μ*M STAT3 antisense (S3AS) but not scrambled nonspecific oligonucleotides (CtrlAS). Additionally, EGF-induced motility was determined in the presence of the EGFR inhibitor PD153035 (PD15). (**B**) The migration distance of DU145WT and PC3 prostate tumour cells after treatment with 1 nM EGF for 24 h was significantly greater and this increase was suppressed by STAT3 siRNA (S3Si) but not siRNA against eGFP (CtrlSi). Shown are mean±s.e.m. for at least three experiments each performed in triplicate. ^*^P<0.01 compared to EGF treatment. The migration distance (on the Y axis) is normalized to diluent alone (NoTx) within each experiment.

EGFR signalling drives motility in fibroblasts and carcinoma cells (Pedersen *et al*, 2004) and invasion of human prostate carcinoma cells (Xie *et al*, 1995; Mamoune *et al*, 2004). Not surprisingly, sustained motility also requires *de novo* RNA synthesis (Chen *et al*, 1994). As such, we asked whether STAT3 might be critical for sustained motility and invasion in response to EGFR signalling. To quantitate cell motility, an *in vitro* wound healing assay was performed and demonstrated that the addition of STAT3 antisense oligonucleotides and the EGFR signalling pathway inhibitor PD153035 (Figure 2A) or siRNA-mediated downregulation (Figure 2B) greatly decreased the migration distance of NR6WT fibroblast cell and DU145WT and PC3 human prostate cancer cells. The extent of migration inhibition was similar to that achieved by blocking EGFR kinase activity with the selective agent PD153035. Interestingly, the level of migration achieved with STAT3 downregulation, and with PD153035 was below that of basal motility, strongly suggesting that either EGFR autocrine signalling via STAT3 or basal STAT3 was contributing to this cell behaviour, as has been implication earlier for EGFR autocrine activation (Xie *et al*, 1995).

**Figure 3:**
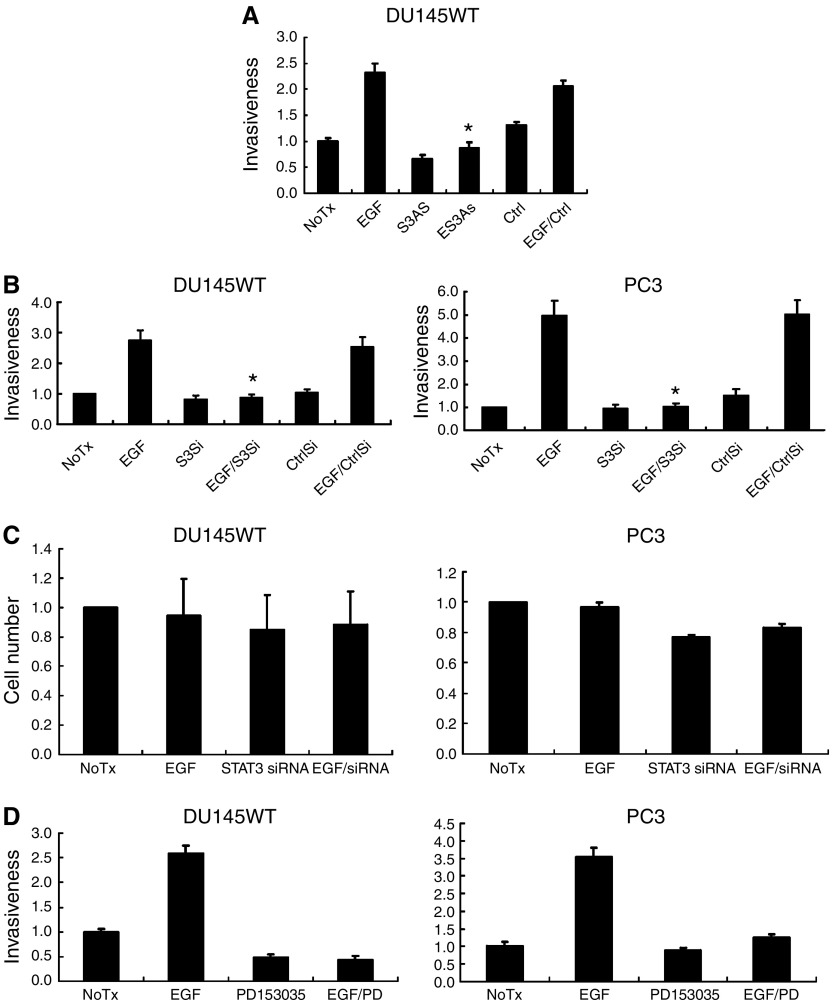
STAT3 is critical for EGFR-mediated prostate tumour cell invasiveness. (**A**) STAT3 antisense (S3AS)-treated DU145WT cells have a much lower invasion rate than those cells treated by EGF. (**B**) STAT3 siRNA (S3Si) inhibited EGFR-mediated invasion of both DU145WT and PC3 prostate tumour cells, whereas eGFP-directed siRNA (CtrlSi) did not. (**C**) STAT3 siRNA downregulation did not significantly affect the number of DU145WT and only somewhat reduced the number of PC3 human prostate cancer cells after 48 h. However, this small decrement, if any, is minor compared to the effect of such treatments on invasion. (**D**) PD153035 (1 *μ*M) blocked the EGF-induced invasiveness of the prostate carcinoma cells. For all experiments shown are mean±s.e.m. for at least three experiments each performed in triplicate. ^*^P<0.05 compared to EGF treatment. The invasiveness as determined by cell number transmigrated through the Matrigel (on the Y axis) is normalized to diluent alone (NoTx) within each experiment.

As motility is a key component to tumour cell invasion (Wells, 2000), we determined whether this inhibition affected tumour cell invasion by examining the transmigration of a Matrigel barrier. Both antisense oligonucleotides and siRNA directed against STAT3 limited tumour cell transmigration to below the levels noted in the absence of added EGF (Figure 3). This extent of inhibition is expected as both DU145WT and PC3 cells express EGFR ligands (Maygarden *et al*, 1994), which generate autocrine stimulatory signals in the physical confines of a matrix, and Matrigel contains competent levels of EGFR ligands (Yudoh *et al*, 1994). That this is due to EGFR signalling is shown by blockade using the selective inhibitor PD153035 (Figure 3C). This inhibitor reduced not only EGF-enhanced invasiveness but also the invasiveness in the absence of exogenous EGF. Thus, during transmigration of Matrigel the cells are in an active EGFR signalling mode.

STAT3 blockade has been found to lead to apoptosis in a number of carcinoma cell lines; such a situation would confound our invasiveness studies. During the migration studies, we did not note such effects morphologically. However, this was tested directly for effects on cell number (Figure 3C). Downregulation of STAT3 using siRNA did not reduce the number of DU145WT cells and only slightly the PC3 cells. In parallel studies, we did not note apoptosis in the absence of external stressor signals (data not shown). Thus, studies on cell migration and invasiveness were not confounded by changes in cell number due to STAT3 downregulation.

### STAT3 signalling is not sufficient for motility

**Figure 4:**
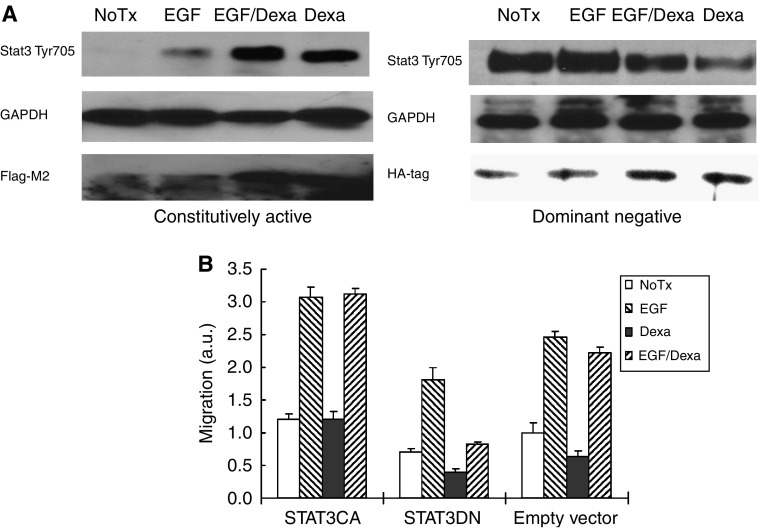
STAT3 is not sufficient for EGFR-mediated migration in NR6WT fibroblasts. (**A**) Immunoblotting demonstrates expression of STAT3 DN and CA mutants in NR6WT cells upon transcriptional upregulation by dexamethasone. Shown are representative blots of at least three experiments each. (**B**) The STAT3 CA mutant did not drive motility of NR6WT cells in the absence or presence EGF, while the DN construct blocked both basal and EGF-induced motility, which indicates STAT3 is critical but not sufficient for the motility. Shown are mean±s.e.m. for at least three experiments each performed in triplicate. ^*^P<0.01 compared to EGF treatment. The migration distance (on the Y axis) is normalized to diluent alone of the empty vector (NoTx) within each experiment.

The foregoing data demonstrate that STAT3 activity is required for increased cell migration and invasion upon EGFR signalling. Upregulation of STAT3 levels in various cancers (Garcia *et al*, 1997; Bromberg *et al*, 1999; Bowman *et al*, 2000; Song and Grandis, 2000) might even suggest this functions as an ‘oncogene’ or ‘tumour progression’ gene. Thus, we asked whether upregulation was sufficient to drive these behaviours. A constitutively active (CA) STAT3 mutant failed (Kijima *et al*, 2002) to drive motility of NR6WT cells in the absence EGF (Figure 4). A DN STAT3 construct did block EGF-induced motility, as expected. The STAT3 constructs were induced by dexamethasone to avoid adaptation or cellular modifications during prolonged selection; the dexamethasone by itself had only a small negative effect on migration that was minimal compared to the effects of either EGF or the constructs. These data are consistent with STAT3 being required, but not sufficient, for transcriptional activation of either replacement proteins or modulation of the proteome at the transcriptional to enable a locomotive state.

### STAT 3 is required for EGFR-mediated expression of VASP and caspase 3

**Figure 5:**
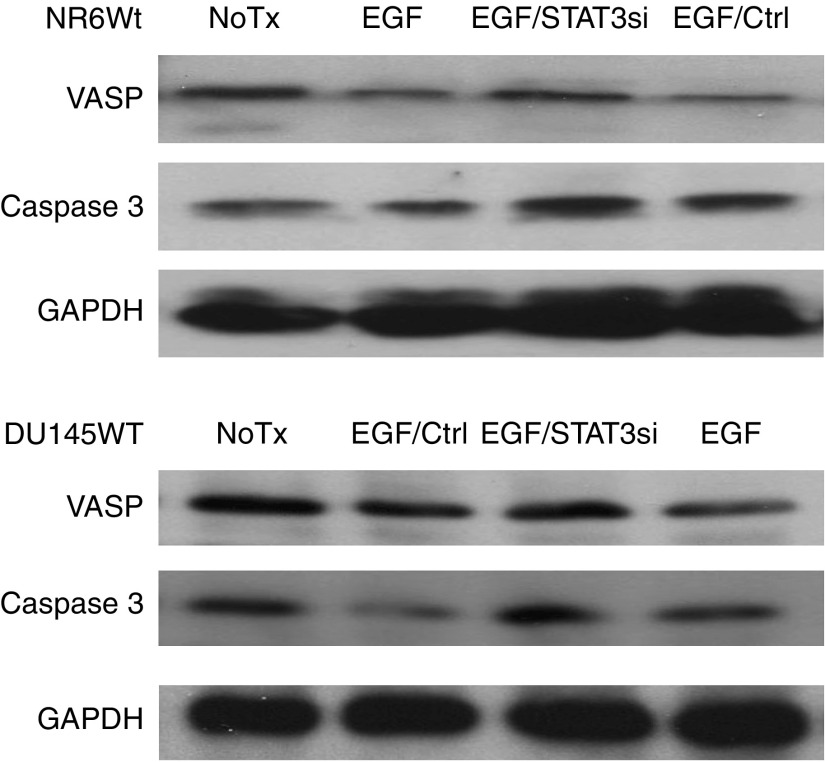
EGF inhibits the expression of VASP and caspase 3 in both NR6WT fibroblast cells and DU145WT human prostate tumour cells and STAT3 siRNA recovered the expression of those two proteins back to a normal level.

STAT3 is well established as a transcription factor that alters the global cell expressome. To further explore which proteins may involve in the EGFR-STAT3 signalling pathway, we performed a microarray analysis in NR6WT cells treated with EGF and/or STAT3 antisense oligonucleotides. The mRNA species that underwent STAT3-dependent, EGF-induced changes included some intriguing targets (Supplemental Data). EGF downregulated ena/VASP, which is a negative regulator of fibroblast cell motility, (Bear *et al*, 2000). EGF also reduced the expression of caspase 3, a central effector of apoptosis. The EGF-induced downregulation of both proteins was abrogated by siRNA treatment of both NR6WT and DU145WT cells (Figure 5). While these studies are just an initial exploration of the underlying mechanisms of STAT3 effects in prostate carcinoma cell invasiveness, they do suggest some molecular effectors.

## DISCUSSION

Carcinogenesis is a multistep process, in which cancer cells disseminate from a localised primary tumour mass to both invade adnexa and metastasize to distant organs. It has been well known that tumour invasiveness is a distinct character of tumour progression involving induced motility in addition to dysregulated proliferation. As there's no catholicon for the current anticancer treatments, disrupting or at least inhibiting the tumour invasiveness would greatly ameliorate the morbidity and mortality. In this study, we investigated whether activated STAT3 protein is involved in the EGF-receptor-mediated migration in fibroblasts and human prostate cancer cells and the invasiveness of the latter. EGF increased STAT3 activity along with cell motility and invasiveness of these cells. Decreased expression and activation of STAT3 by treatment with STAT3 antisense oligo nucleotides or siRNA or in the presence of a DN mutant of STAT3 abrogated migration and invasion. The results together, indicated that STAT3 could be a targeted critical element in the EGF-mediated cell migration and invasion of prostate carcinoma cells.

Epigenetic events, in addition to transcriptome changes signalled through STAT3, also contribute to tumour cell motility and invasion. Comparing the migration of NR6WT cells expressing constitutively activated STAT3 to those expressing the STAT3 DN mutants, it is interesting to find that constitutively activated STAT3 alone does not increase cell motility, nor does it increase EGF-stimulated migration. This result suggests that STAT3 is not sufficient for cell migration but that it provides for end effectors that require additional signals for either activation or full phenotypic expression. EGF stimulation of a cell results in the activation of multiple pathways that lead to transcriptional control including Src, PLC*γ*, PI3-K, Ras/MAPK and JAK/STAT (Wells, 1999). These pathways are often functionally interlinked (Jorissen *et al*, 2003). Thus, the need for *de novo* transcription and translation that one notes during growth factor-induced motility (Kislauskis *et al*, 1997; Mingle *et al*, 2005), can be accomplished by a number of downstream transcription factors but requires STAT3 in addition to the previously determined PLC*γ* pathway (Manos *et al*, 2001; Mamoune *et al*, 2004). Recently, it has been noted that EGFR signalling promotes progression of T24 bladder carcinoma cells via STAT3 upregulation of transcription, including that of matrix metaloproteinases (Itoh *et al*, 2006).

To further probe the role of the EGF/STAT3 pathway, an initial microarray analysis was performed on NR6WT cells, thus avoiding variable amounts of autocrine signalling, to determine transcripts that were altered by EGF in a STAT3-dependent manner. (The subset of these transcripts that are classified as related motility and cell adhesion are provided in the Supplemental Table.) Interestingly, these did not include the constituents of the well-known epigenetic cascades but rather extracellular matrix or cell cytoskeleton components. It is possible that the enzymatic epigenetic effects are regulated by the other EGFR-induced transcription cascades, and that STAT3 primarily functions to provide the motors and pathways for motility. Of note, we did not detect either collagenase-1 (MMP-1) or stromelysin-2 (MMP-10) that was seen to be upregulated in T24 bladder carcinoma cells (Itoh *et al*, 2006); this discrepancy is likely due to our examining the nontransformed NR6WT fibroblasts presenting a more limited set of transcription changes, although the two changes noted in the fibroblasts also were found in the cancer cells (Figure 5). Obviously, these early hints are under active further investigation as these questions lie beyond the scope of the current communication.

Recent studies suggest that aberrant STAT3 signalling may play an important role in the carcinogenesis of prostate cancer. Previous data from other groups shows higher phospho-STAT3 expression in prostate tumour tissues than in adjacent normal tissues (Dhir *et al*, 2002; Mora *et al*, 2002), and that this correlates with invasiveness (Horinaga *et al*, 2005). We also found increased expression of STAT3 protein in prostate tumour tissues compared to normal tissue from the same patient (data not shown, as similar data have been previously published). It is quite reasonable that the high expressed pSTAT3 in prostate tumour tissues is from the increased expression of STAT3 as well as activation by autocrine growth factor signalling (Song *et al*, 2001). Our study indicating that STAT3 is critical for invasion of prostate cancer cells DU145WT and PC3, secondary to increased cell motility is consistent with these findings and provide for a mechanism by which pSTAT exerts its effects.

Contrary to other studies (Barton *et al*, 2004; Pedranzini *et al*, 2004), we did not find decreased tumour cell numbers upon STAT3 downregulation (Figure 3); this may be due to the incomplete nature of such interventions (although the other studies suffered from similar nonquantitative abrogation), high levels of autocrine EGFR signalling overcoming this limitation via other pathways, or likely the shorter time course of our experiments. However, in accord with these other studies, when we overexpressed the DN STAT3 construct in DU145WT cells, we noted a high degree of apoptosis in these cells (for the experiments in Figure 4, we achieved a lower, non-apoptotic level of DN STAT3); our failure to established stable clones expressing this DN construct likely relates to this inhibition of proliferation and/or increased apoptosis. In addition, when we stress the cells using apoptosis inducers the blockade of STAT3 does increase tumour cell apoptosis. As such, we feel that this discrepancy is more likely a quantitative rather than a qualitative effect.

In sum, the model which emerges is one in which STAT3 signalling downstream from EGFR is required for persistent cell motility and invasion, and that partial abrogation of this pathway hinders this tumour progression. Only more extensive abrogation of STAT3 signalling compromises carcinoma cell survival and proliferation. Thus, STAT3 inhibition, even if suboptimal, would slow tumour progression.
