# Supplementary material for: STAT3 is required but not sufficient for EGF receptor-mediated migration and invasion of human prostate carcinoma cell lines
Source: Br J Cancer. 2006 Jun 27;95(2):164–71. doi: 10.1038/sj.bjc.6603234 (PMC2360627; doi:10.1038/sj.bjc.6603234)
Supplement: Supplemental Table Information [file 95-6603234x2.doc]

Supplemental Table. Transcript levels altered by EGF in a STAT3-dependent manner in NR6WT cells. A microarray analysis was performed using the Affymetrix chip U74AV2 for murine transcripts. Shown are transcripts whose levels were altered in the presence of EGF (1nM for 24 hr) at a level considered statistically significant by whole transcript analysis and reverted to baseline in the present of STAT3 antisense (10uM). Shown are only those transcripts considered to provide for cell motility and adhesion. The alteration in levels of a small number of these transcripts have been validated by immunoblotting of the proteins. The two columns describe the level change in the presence of EGF compared to diluent alone, and then the reversion towards baseline in the EGF when STAT3 has been downregulated.
